# Being human is a gut feeling

**DOI:** 10.1186/s40168-015-0076-7

**Published:** 2015-03-13

**Authors:** Thiago Hutter, Carine Gimbert, Frédéric Bouchard, François-Joseph Lapointe

**Affiliations:** Département de philosophie, Université de Montréal, P. O. Box 6128, Station Centre-ville, Montréal, QC H3C 3J7 Canada; Département de sciences biologiques, Université de Montréal, P. O. Box 6128, Station Centre-ville, Montréal, QC H3C 3J7 Canada; Institut national des sciences appliquées de Lyon, 20 Avenue Albert Einstein, 69621 Villeurbanne CEDEX, France

**Keywords:** *Homo sapiens*, Functional integration, Biological individuality, Super-individual

## Abstract

Some metagenomic studies have suggested that less than 10% of the cells that comprise our bodies are *Homo sapiens* cells. The remaining 90% are bacterial cells. The description of this so-called human microbiome is of great interest and importance for several reasons. For one, it helps us redefine what a biological individual is. We suggest that a human individual is now best described as a super-individual in which a large number of different species (including *Homo sapiens*) coexist. New concepts of biological individuality must extend beyond the traditional limitations of our own skin to include our resident microbes. Besides its important contributions to science, microbiome research raises philosophical questions that strike close to home. What is left of *Homo sapiens*? If most of our cells are not *Homo sapiens* cells, what does it mean to be an individual human being? In this paper, we argue that the biological individual is determined by the amount of functional integration among its constitutive parts, a definition that applies perfectly to *Homo sapiens* and its microbiome.

## Background

In *the Origin of Species*, Darwin posits that the process of natural selection directly acts on species by affecting the reproductive success and survival of the individuals constituting them. But what exactly are these individuals? Intuitively, we tend to think that the entities upon which selection acts are what common sense would describe as an organism, for example, a dog, a cow, and a human being. However, recent findings in various domains of biology such as physiology [1-3], sociobiology [4], microbiology [5], metagenomics [6], immunology [7], as well evolutionary transitions [8] have brought into question our intuitions concerning the correspondence between our notions of *individual* and *organism* by showing the tensions that arise between these two concepts. Such concerns have been in the heart of heated disputes in philosophy of biology [9-13], but they have not yet fully penetrated the scientific community.

By emphasizing the relation that humans have with their microbiomes, we aim to question the definition of human individuality in biological research by showing that the entity that we traditionally conceive as the organism called ‘human being’ is not the individual we intuitively think it is. There are many ways to approach the concept of biological individuality. Here, we will focus on one particular approach in order to raise the question of individuality in humans. By focusing on the biological aspect of individuality and its relation to fitness, we distance ourselves from philosophical questions concerning selfhood and personal identity [14]. Our claim is that, with respect to most biological research projects, human beings are so well integrated with their microbiomes that the individuality of human beings is better conceived as a symbiotic entity. Insofar as biological research is concerned, to be human is to be multispecies.

## Main text

Most notions of individuality in biological research have directly or indirectly called upon evolutionary considerations, but why is that? Our understanding of individuality (be it an individual chair or an individual giraffe) has historically been linked to the question of organization: an individual has often been conceived as an organized whole, distinguishing it from a mere collection of disjointed parts. For artifacts such as individual chairs, the origin of organization was easier to establish given the clear human intentionality found in the design of the artifact (that is, a chair is a functional whole that has a specific purpose because we designed and built them to have that integrated function); for biological individuals however, the question was much more complicated. Before Darwin, intelligent design arguments (such as the ones found in Paley) explaining the organization found in biological individuals via divine creation were the norm. Since Darwin, the origin of organization of biological individuals is to be explained thanks to designer-free adaptive processes. Individuals were functional wholes whose parts-integration was the result of evolution by natural selection.

One of the advantages of focusing on evolutionary considerations is that it also allows to account for collectives of individual organisms acting as emergent super-individuals, such as bee colonies being recognized as what is often referred to as ‘super-organisms.’ As suggested by E. O. Wilson [15], the question of individuality emerging from functional integration can be read in the following way: when the behaviors of members of a society - or a group - become so well organized, coordinated, and integrated that the degree of functional organization approaches - or rivals - that of the integration between the parts of an individual organism, is it still truly a group of singular beings? In fact, when such a group of entities acquires such a high degree of organization, it may become fitness-bearing in the right way and thus be defined and recognized as a proper unit of selection above and beyond the individual organisms forming the group [16]. If biological individuality is to be conceived as being an evolutionary individual, the unit of selection debate will intersect with our understanding of individuality when the unit of selection achieves higher fitness thanks to higher functional integration. Here, we side with the position asserting that the functions accomplished by integrated entities are the result of collaboration between diverse entities [17]. This collaboration is sometimes between members of a same species (for example, bees) or, more controversially, between members of different species. If individuality is about organization, and if that organization in the case of biological individuals emanates from evolution by natural selection, one needs a revised account of fitness to account for the emergence of multispecies individuals.

One of the problems of accounting for the functional integration of distinct individual organisms into an emergent super-individual is that it is not obvious how to aggregate the evolutionary success (or adaptedness) of individuals with autonomous evolutionary histories. One cannot readily compare or add up the reproductive success (fitness in the traditional sense) of organism X from species A to that of organism Y from species B. Interspecific fitness comparisons are usually frowned upon for that reason. Figuring out how to identify the degree of fitness alignment between organisms of different species requires the identification of a common evolutionary currency that is distinct from reproductive success. Alternative measures of fitness such as energy control [18] or differential persistence [19-21] have been suggested to allow for interspecies fitness comparisons and for fitness attributions to multispecies community level individuals. We favor the latter to account for multispecies assemblages such as the individuals that emerge from symbiotic interactions, because persistence is a necessary aspect of functional integration, whereas such integration could be achieved without fluctuations of energy control. Furthermore, the concept of persistence can also account for individuals emerging from multiple species interacting via abiotic parts of the environment [22]. In this respect, our account of biological individuality differs from others proposed in biology [23,24]. However, fully explaining this theory and its implications lies beyond the scope of this paper.

## Discussion

How do these issues illuminate our understanding of our own biological individuality? It is common knowledge that bacterial presence is ubiquitous in every single surface of the environment exterior to any single organism and inside of it. Human beings are in constant interaction with the bacterial world, whether at the interface of their epidermis or through their digestive tract. If individuality is simply characterized by the amount of integration, the interaction between humans and microbes allows us to raise one important question: is the organism currently recognized as a human being the real individual? Indeed, the different sites of the human body are inhabited by millions of bacterial cells - the so-called *human microbiome* - interacting with each other as well as with human cells [25,26]. Such bacterial communities differ in diversity and proportion according to specific body habitats [27], which in turn ensures that each localized microbiome accomplishes different functions that affect a person’s health and well-being. Namely, the gut microbiome, constituted mostly by *Bacteroidetes* and *Firmicutes*, is involved in the fermentation that enables bacteria to live in an anaerobic environment [28]. This process is used to produce short-chain fatty acid through the conversion of sugars, which are used by human cells as a source of energy. Metabolic activities of the gut microbiome also increase the amount of indispensable amino acids (that is, lysine) and contribute to the degradation of xenobiotics such as benzoate, a common food supplement involved in the biosynthesis of B9 and B12 vitamins [29]. Therefore, the bacterial communities inhabiting the human gut are an essential component of human digestion [30], and the corresponding intestinal microbiome is as important as a functional heart or kidney for survival [23]. In many respects, our microbiome-based digestion is more essential to the survival of an individual than the maintenance of other organs. In this light, being a human biological individual is to be a community of *Homo sapiens* and microbial symbionts whose degree of functional integration (and degree of individuality) is a function of the potential of that community to persist and evolve as a whole.

Because all living organisms (human beings included) rely so heavily on their microbiome to perform some of the metabolic functions that keep them alive, we claim that this symbiotic association is bound by a common evolutionary fate. The idea of *common fate* utilized in some accounts of biological individuality reflects the notion that the functional whole that we define as the organism and its microbiome can stand or fall as a whole when undergoing a selective pressure [16,22]. One notable example of such an evolutionary process is illustrated by the detrimental effects of *Clostridium difficile* on the functionality of the gut microbiome [31] and survival in humans [32]. At a higher level, one can also pinpoint the effect of the microbiome on reproductive success (and fitness) among closely related species and the role that gut bacteria play in speciation [33,34]. That is that, it is the sum of an organism’s genome and microbiome - the *hologenome* - and the processes they make possible that are linked by a common evolutionary fate (extinction, speciation) and selected together as a whole [35,36].

## Conclusions

If individuality is a matter of being functionally integrated to the extent that the causality between the interacting parts may persist or cease when one of these parts is faced with an evolutionary pressure, we ought to consider, for biological research purposes, that the single *Homo sapiens* is not in fact the real biological individual. While a simpler mono-species view of individuality may be sufficient for most of our everyday social interactions, the real *biological* individual is a *super-individual* defined as the sum of the organism + its microbiome; it is this *integrated symbiotic association* that is able to persist and survive. As the poet Walt Whitman aptly pointed out in *Song of Myself*, ‘I am large, I contain multitudes.’
